# The C_2_N surface as a highly selective sensor for the detection of nitrogen iodide from a mixture of NX_3_ (X = Cl, Br, I) explosives†

**DOI:** 10.1039/d0ra04930a

**Published:** 2020-08-27

**Authors:** Muhammad Yar, Muhammad Ali Hashmi, Khurshid Ayub

**Affiliations:** Department of Chemistry, COMSATS University, Abbottabad Campus KPK 22060 Pakistan khurshid@cuiatd.edu.pk +92-992-383591; Department of Chemistry, University of Education, Attock Campus Attock 43600 Punjab Pakistan

## Abstract

Explosives are quite toxic and destructive; therefore, it is necessary to not only detect them but also remove them. The adsorption behavior of NX_3_ analytes (NCl_3_, NBr_3_ and NI_3_) over the microporous C_2_N surface was evaluated by DFT calculations. The nature of interactions between NX_3_ and C_2_N was characterized by adsorption energy, NCI, QTAIM, SAPT0, NBO, EDD and FMO analysis. The interaction energies of NX_3_ with C_2_N are in the range of −10.85 to −16.31 kcal mol^−1^ and follow the order of NCl_3_@C_2_N > NBr_3_@C_2_N > NI_3_@C_2_N, respectively. The 3D isosurfaces and 2D-RGD graph of NCI analysis qualitatively confirmed the existence of halogen bonding interactions among the studied systems. Halogen bonding was quantified by SAPT0 component energy analysis. The SAPT0 results revealed that Δ*E*_disp_ (56.75%) is the dominant contributor towards interaction energy, whereas contributions from Δ*E*_elst_ and Δ*E*_ind_ are 29.41% and 14.34%, respectively. The QTAIM analysis also confirmed the presence of halogen bonding between atoms of NX_3_ and C_2_N surface. EDD analysis also validated NCI, QTAIM and NBO analysis. FMO analysis revealed that the adsorption of NI_3_ on the C_2_N surface caused the highest change in the *E*_HOMO–LUMO_ gap (from 5.71 to 4.15 eV), and resulted in high sensitivity and selectivity of the C_2_N surface towards NI_3_, as compared to other analytes. It is worth mentioning that in all complexes, a significant difference in the *E*_HOMO–LUMO_ gap was seen when electronic transitions occurred from the analyte to the C_2_N surface.

## Abstract 2

Explosives are quite toxic and destructive; therefore, it is necessary to not only detect them but also remove them.

## Graphical abstract

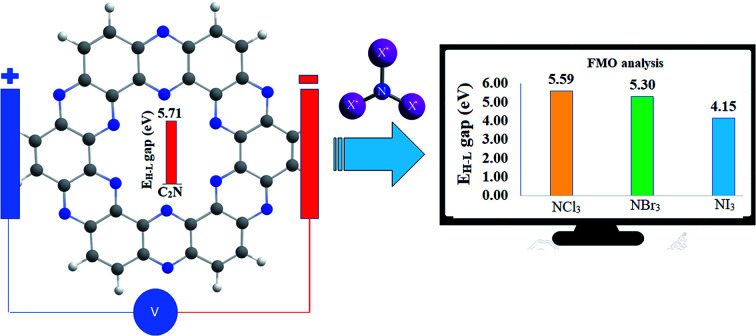

## 1. Introduction

Chemical warfare and toxic explosives pose serious threats to civilians and the military defense system. It is essential to quickly detect and dispose of these chemical warfare agents (CWAs) and toxic explosives for the safety and security of civilian and military defense systems. Nitrogen halides are extremely explosive materials in which the N-atom is connected with one of the halogen atoms (Cl, Br and I). NI_3_ is a highly shock-sensitive red crystalline material and can even explode under slight mechanical stress.^1,2^ It is usually stable in NI_3_·NH_3_ adduct form. NCl_3_ is a bright yellow volatile liquid that has an unpleasant smell, with a reported melting point of −27 °C and can auto-ignite in the range of 71–73 °C.^3^ The red oily volatile liquid NBr_3_ is also a temperature-sensitive agent.^4^ The literature review revealed that many studies have been carried out for the detection of NCl_3_ on different surfaces with different chemical methods.^5–8^ As far as we know, no such studies have been reported for NBr_3_ and NI_3_ due to their extreme explosive nature.

In recent times, researchers have been mainly working to develop a method for the disposal and detection of these explosives.^9,10^ For the detection and decay of these explosives, various expensive techniques, such as gas and liquid chromatography^11^ and spectroscopic techniques have been applied; however, these techniques fail to detect these explosives at low concentrations.^12^ Moreover, these techniques also require sophisticated sample preparation and skilled persons. The prime requisites for a quality sensor are low cost, fast response, metal-free nature, high selectivity and reproducibility.^13,14^ Moreover, it should have a high surface to volume ratio over which the analyte can easily and effectively interact.^15^

A large number of materials such as zeolites,^16^ metal–organic frameworks,^2,3,17,18^ metal clusters,^19^ pristine and boron-doped graphene,^20^ graphene,^21–23^ semiconductor nanowires,^24^ carbon nitride,^25^ boron nitride,^26^ aluminum nitride^27^ and phosphorous carbide sheets,^28^ have been studied in the past few decades as sensors for detecting trace quantities of these explosives. The major problems still faced by the sensor industries are cost, limited sensitivity, and the lack of reproducibility and sensitivity of the sensor to humidity and temperature.

In this study, we selected the electron-rich nitrogenated holey graphene (C_2_N) surface as the electrochemical sensor for the detection and capture of nitrogen halide (NCl_3_, NBr_3_ and NI_3_) explosives. In the recent past, several studies have been devoted to predicting the stabilities of yet to be synthesized 2D surfaces. For example, Gueorguiev and coworkers studied the stability of CFx surfaces through first-principles calculations. In another study, the effects of hydrogenation and fluorination in curved 2D carbon surfaces were explored.^29,30^ The C_2_N is a 2D material with controlled pore size and periodic geometry. It was recently synthesized by Mahmood *et al.*, *via* wet chemistry.^31^ C_2_N consists of fused rings of benzene and pyrazine, which make it a highly π-conjugated structure. C_2_N has been used in many fields like batteries,^32–34^ catalysis,^35,36^ optical devices,^37^ gas storage^38,39^ and photo-catalysis,^40^ C_2_N surface with electron-rich nitrogenated cavity has tremendous potential of electrochemical sensor, as reported earlier by Xu *et al.*^41^ It has also been studied for physical adsorption of toxic and noble gases like H_2_S, HF, NH_3_, HCN, CH_4_, N_2_, CO_2_, He, Ne and Ar.^42,43^ To the best of our knowledge, the selected C_2_N surface has not been previously studied as an electrochemical sensor for NX_3_ explosives. The electron-rich nitrogenated cavity of C_2_N is thermally stable with a high surface to volume ratio, which provides an ideal environment for the interaction of analytes. Density functional theory (DFT) calculations were executed to investigate the adsorption of NX_3_ on the C_2_N surface. The nature of the non-covalent interactions between NX_3_ and the C_2_N surface was further explored through Bader's quantum theory of atoms in molecules (QTAIM), non-covalent interactions (NCI), and symmetry adopted perturbation theory (SAPT0) analysis. The electronic properties of NX_3_@C_2_N complexes were studied through electronic density differences (EDD), natural bond orbital analysis (NBO) and frontier molecular orbital analysis (FMO).

## 2. Computational methodology

All DFT calculations for the adsorption of NX_3_ analytes on C_2_N surface were executed using the Gaussian 09 software due to its established accuracy and efficacy. For the geometry optimization of analtyes@C_2_N complexes, the M05-2X/LANL2DZ level of theory was applied, which is the best for studies of non-covalent interactions.^44–53^ The level of theory chosen was quite adequate for such a complex system. The literature revealed several examples where complex systems were treated with a comparable level of theory.^47,50,54–56,58^ The non-bonding interactions can play a vital role in deciding the properties of a system and need to be modeled accurately to determine the interactions of NX_3_ analytes with the C_2_N surface. Although NI_3_ exists in the form of the NI_3_·NH_3_ adduct, we did not include this adduct in our study because we wanted to have a comparative study of NX_3_ explosives. This adduct is important only for NI_3_ but not for other NX_3_ molecules. The visualization of the geometries and the evaluation of the structural parameters were carried out using the GaussView 5.0 ^57,59,60^ and Chemcraft software.^61^ To confirm the optimized geometries as true minima on the potential energy surface, vibrational frequency analysis was performed at the same level of theory.

In search of stable geometries containing the analytes@C_2_N complex, many different possible orientations of each analyte on the C_2_N surface were simulated. The adsorption energies of analytes@C_2_N surface are defined as follows:

1 Δ*E* = [*E*_NX_3_@C_2_N_ − (*E*_NX_3__ + *E*_C_2_N_)]

where *E*_NX_3_@C_2_N_, *E*_NX_3__ and *E*_C_2_N_ are the energies of the complex, analytes and C_2_N, respectively. The adsorption energies of stable geometries for these complexes were further corrected by the counterpoise method to avoid basis set superposition error (BSSE). The BSSE corrected energy is calculated as follows:

2 *E*_int.CP_ = *E*_int_ − *E*_BSSE_

where *E*_int.CP_, *E*_int_ and *E*_BSSE_ are the counterpoised corrected energy, non-corrected interaction energy and basis set superposition error energies of NX_3_@C_2_N complexes.

Symmetry adapted perturbation theory (SATP0) analysis was performed with the PSI4 software.^51^ SAPT0 analysis gives a quantitative idea about the components of energies (induction, dispersion, exchange and electrostatic) involved in non-covalent interactions between NX_3_ and the C_2_N surface. The total energy of SAPT0 components is represented as follows:

3 Δ*E*_int_ = Δ*E*_elstat_ + Δ*E*_exch_ + Δ*E*_ind_ + Δ*E*_disp_

Induction energy (Δ*E*_ind_) arises due to interactions between filled and partially filled orbitals, while the exchange (Δ*E*_exch_) energy part of SAPT0 indicates repulsion between the two fragments of filled orbitals. Similarly, the dispersion energy (Δ*E*_disp_) term represents attractive interactions between filled orbitals where the electrostatic energy (Δ*E*_elst_) part arises due to interactions between polarized orbitals of two fragments.

Non-covalent interactions between NX_3_ and C_2_N were mapped using Multiwfn 3.7 software.^62,63^ NCI analysis is described by two factors, *i.e.*, the electron density (*ρ*) and reduced density gradient (RGD), for understanding the nature of the interactions. The relationship between these factors is represented as follows:4
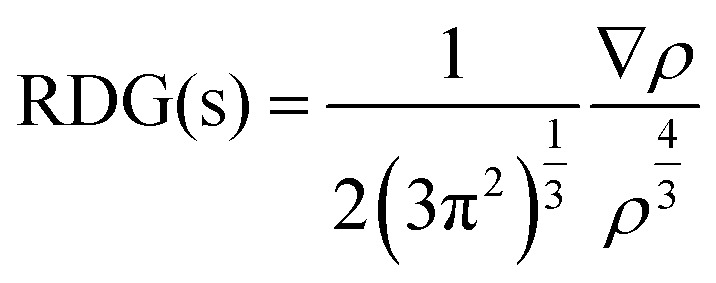


The sign and the value of the electron density (*ρ*) is a major descriptor for the characterization of the nature of non-covalent interactions. The plot of RDG *versus* (*λ*_2_)*ρ* signifies the nature of the interactions. Here, *λ*_2_ is the second Eigenvalue of the Hessian matrix, which gives better information for understanding the nature of interactions. The sign (*λ*_2_)*ρ* < 0 represents the accumulations of the electron density (non-covalent interactions), while the sign (*λ*_2_)*ρ* > 0 represents the depletion of the electron density (repulsive interactions). The signs and values of (*λ*_2_)*ρ* are also represented in color-coded form, which are green ((*λ*_2_)*ρ* < 0 to −0.01 a.u.), red ((*λ*_2_)*ρ* > 0) and blue ((*λ*_2_)*ρ* < −0.01 a.u.) to show weak van der Waals, repulsive and H-bonding interactions, respectively.^64,65^

Non-covalent interactions were further quantified by Bader's quantum theory of atoms in molecules (QTAIM).^66,67^ The topological parameters of QTAIM analysis used in non-covalent interactions are electron density (*ρ*), the Laplacian (∇^2^*ρ*), −*V*/*G*, energy density (*H*(*r*)) and *E*_int_ (individual bond interactions energy). The non-covalent interactions between interacting systems in QTAIM analysis are usually predicted by the bond critical point.^68^ Besides the above-mentioned interaction properties, we also studied the charge transfer of NX_3_@C_2_N complexes through electron density differences (EDD)^69–71^ and natural bond orbitals (NBO). EDD analysis was conducted by taking the difference in the electron density of the complex from its constituents (C_2_N and analyte). Frontier molecular orbital analysis (FMO) was executed to study the changes in the conductivity of the C_2_N before and after complexation with analytes.

## 3. Results and discussion

### 3.1 Geometric optimization and interaction energies

**Fig. 1:**
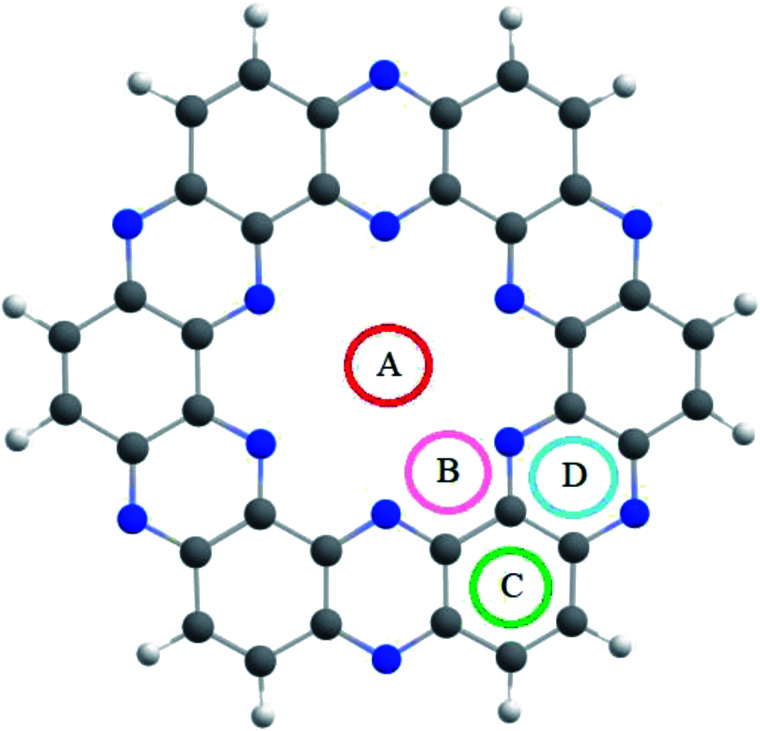
The optimized geometry of the nitrogenated holey graphene (C_2_N) at the M05-2X/LANL2DZ level of theory.

The optimized C_2_N single-layer structure selected as the electrochemical sensor is presented in Fig. 1, which consists of benzene and pyrazine rings in the fused form. The nitrogen atoms present in the pyrazine ring are arranged periodically in such a way that they form an electron-rich nitrogenated cavity with 8.30 Å diameter.^31^ The existence of the electron-rich nitrogenated cavity of the C_2_N surface offers powerful binding and capturing frameworks for shock sensitive agents (NX_3_).

**Fig. 2:**
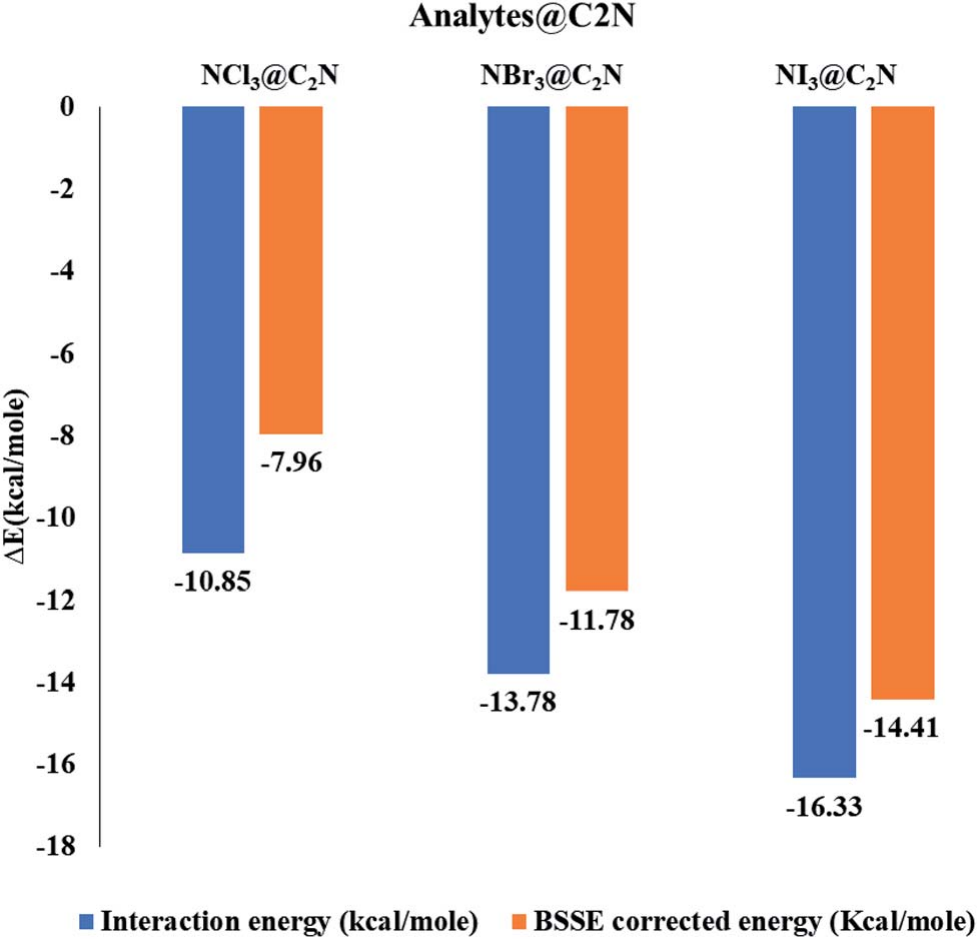
Graphical representation of the interactions and BSSE energies of NX_3_@C_2_N complexes.

The binding sites over the C_2_N structure are represented as A (center of cavity), B (triangle between two nitrogen), C (at top of benzene) and D (at pyrazine rings) (see Fig. 1). To find the stable geometry of the analytes@C_2_N complexes, all possible orientations of each analyte over the four possible binding sites are tested. The most stable optimized geometries of analytes@C_2_N are given in Fig. 2, while the rest of the geometries are shown in Fig. S1 (ESI†).

The interaction energies and bond lengths of the studied analytes are given in Table 1 and Fig. 2 (graphical form). The observed interaction energies of the most stable studied NX_3_@C_2_N complexes are −10.85 kcal mol^−1^ (NCl_3_), −13.78 kcal mol^−1^ (NBr_3_) and −16.33 kcal mol^−1^ (NI_3_), while other interaction energies are given in Table S1.† Similarly, energies of complexes are also corrected by the basis set superposition error (see Table 1).

**Table 1:** Interaction and BSSE energies (kcal mol^−1^) of NX_3_@C_2_N complexes

Bond length (Å)	A	Bond length (Å)	B	Bond length (Å)	C	Bond length (Å)	D	Bond length (Å)	*E*_int_ (kcal mol^−1^)	BSSE (kcal mol^−1^)
NCl_3_@C_2_N	Cl4⋯N1	3.18	Cl4⋯N2	3.00	Cl4⋯N3	3.18	Cl4⋯X	1.84	−10.85	−7.96
NBr_3_@C_2_N	Br4⋯N1	3.22	Br4 ⋯N2	3.01	Br4⋯N3	3.18	Br4⋯X	1.87	−13.78	−11.34
NI_3_@C_2_N	I4⋯N1	3.25	I4⋯N2	3.01	I4⋯N3	3.24	I4⋯X	1.94	−16.33	−14.41

The analytes studied for sensor studies were nitrogen halides. In most of the complexes, the halogen atoms (Cl, Br, I) of analytes interacted with the N-atoms of the C_2_N cavity. Such non-covalent interactions where covalently bonded halogens interact with high electronegative atoms are known as halogen bonding. Halogen bonding is an intermolecular force of attraction in which halogen atoms accept electrons from highly electronegative atoms.^72^ Halogen bonding interactions have been the topic of recent interest in the scientific community because of their potential for use in supramolecular chemistry,^73,74^ crystal engineering,^75,76^ molecular recognition,^77^ rational drug design,^78^ and catalysis.^79^ In these interactions, a part of the halogen acts as the electrophile (σ-hole).^80^ The σ-hole interactions increase as the size and polarizability of the halogen atoms (I > Br > Cl > F) increase.^81^ This trend of halogen polarizabilities shows that the I-atom should form stronger halogen bonds with Lewis bases (N-atoms). The same trend of halogen bonding was observed in our studied complexes (*vide infra*).

The stable conformation of NCl_3_@C_2_N resulted in the adsorption energy of −10.85 kcal mol^−1^ (BSSE = −7.96 kcal mol^−1^). The optimized geometry of the stable NCl_3_@C_2_N complex revealed that one of the Cl atom of NCl_3_ was projected towards the center of the C_2_N cavity (site A) with the interaction distance of 1.84 Å, while the other two Cl atoms moved upwards. The Cl atom of NCl_3_ and N-atoms (site B) of the C_2_N surface interacted through a longer bond distance as compared to site A (see Table 1). However, halogen bonding between the Cl atom of NCl_3_ and N-atoms of the C_2_N produced a stable optimized geometry of the complex.

**Fig. 3:**
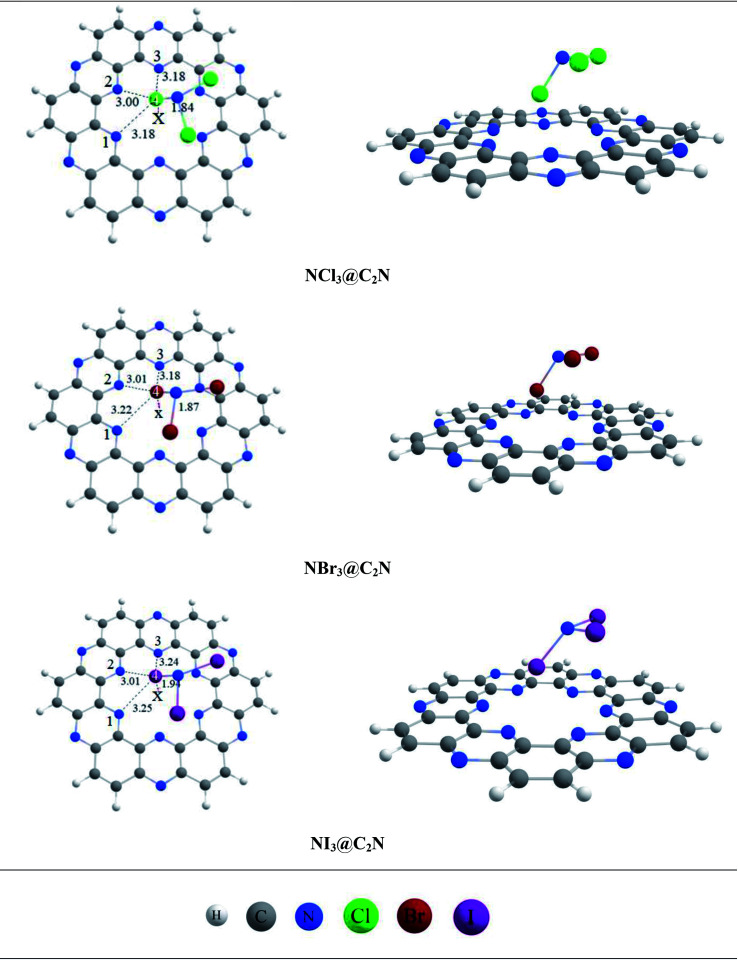
The top and side views of the optimized geometries of analytes@C_2_N at the M05-2X/LANL2DZ level of theory.

The same strategy was adopted for the NBr_3_@C_2_N complex (as with the NCl_3_@C_2_N complex) to obtain a stable geometry. The stable NBr_3_@C_2_N complex had an interaction energy of −13.78 kcal mol^−1^ (BSSE = −11.78 kcal mol^−1^). The interaction energy of NBr_3_@C_2_N was greater than that of the NCl_3_@C_2_N complex. The orientation of NBr_3_ over C_2_N in the stable conformation was similar to that of the NCl_3_@C_2_N complex (Fig. 3). The Br-atom of NBr_3_ interacts at site A of the C_2_N cavity through an interaction distance of 1.87 Å. The interaction distances were in the range of 3.01 to 3.22 Å, through which the other Br-atoms of NBr_3_ interact with the N-atoms of the C_2_N surface.

The stable optimized geometry of the NI_3_@C_2_N complex (*E*_int_ = −16.31 kcal mol^−1^; BSSE = −14.41 kcal mol^−1^) had the same orientation as the other two NX_3_@C_2_N complexes. The I-atom of NI_3_ interacted at site A of the C_2_N cavity at the interaction distance of 1.94 Å. The optimized geometry of the NI_3_@C_2_N complex revealed that the I-atom of NI_3_ interacted with the N-atoms of C_2_N with interaction distances ranging from 3.01 Å to 3.25 Å.

The results of all complexes showed that the X-atoms (halogens) acted as electrophiles (σ-hole) in non-covalent interactions with the electron-rich nitrogenated cavity of C_2_N. The interaction energy results of the NX_3_@C_2_N complexes supported the existence of the physisorption mechanism. The interaction energy trend observed for the NX_3_@C_2_N complexes was NCl_3_@C_2_N > NBr_3_@C_2_N > NI_3_@C_2_N. The interaction energy results indicate that C_2_N can accommodate NX_3_ analytes on its surface, but the highest interaction energy was seen for the NI_3_@C_2_N complexes (−16.31 kcal mol^−1^). The I-atoms of NI_3_ interacted through longer bond distances with the C_2_N surface as compared to the NBr_3_ and NCl_3_. The possible reason for the high interaction energy for the NI_3_@C_2_N complex was the stronger halogen bonding between the I-atoms of NI_3_ and N-atoms of C_2_N.

### 3.2 Non-covalent interaction (NCI) analysis

**Fig. 4:**
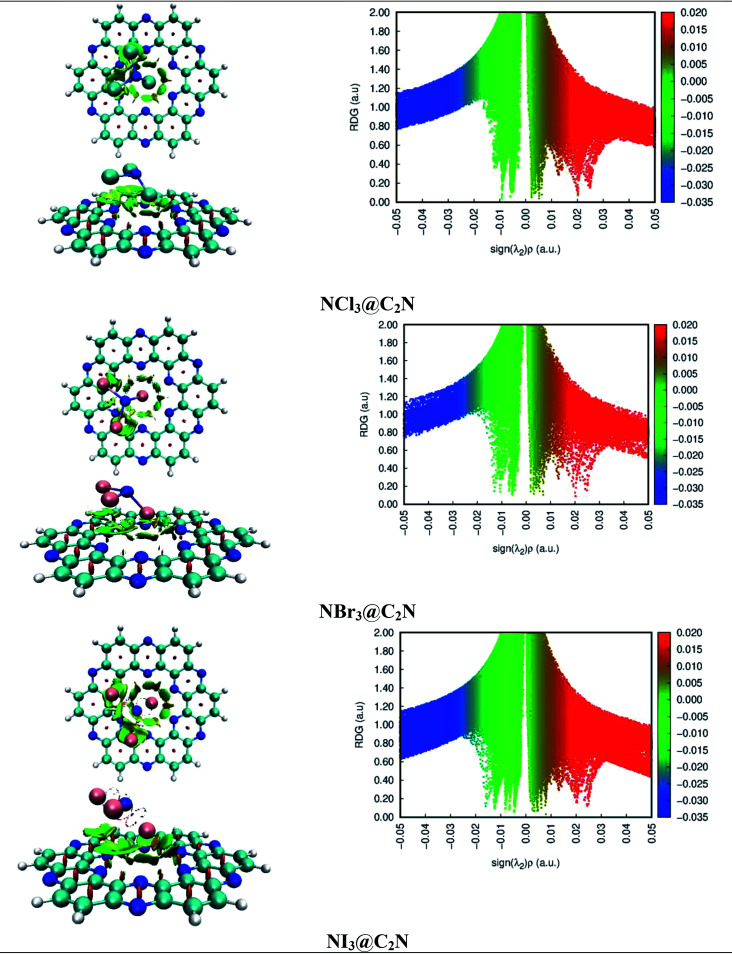
NCI isosurfaces and 2D-RDG graphs of the optimized geometries of stable complexes obtained *via* the M05-2X method (iso value = 0.05 a.u.).

NCI analysis was carried out to understand the nature of the intermolecular interactions. The NCI analysis consists of 3D isosurfaces and 2D reduced density gradient (RDG) graphs of the complexes. The 2D-RDG graph and 3D isosurfaces of the NCI analysis for the NX_3_@C_2_N complexes are presented in Fig. 4. The 3D isosurfaces of all the analytes showed the presence of green patches between the surface of C_2_N and the analytes. Similarly, the red patch projection was also seen in the benzene and pyrazine ring of the C_2_N cavity. For NCl_3_@C_2_N, the 3D isosurface showed the presence of weak dispersive interactions between the Cl atoms of NCl_3_ and N&C-atoms of the C_2_N surface; this can be seen in the 2D-RGD graph. The projection of green spikes appeared between 0 to −0.01 a.u. at the (*λ*_2_)*ρ* axis, which confirmed the existence of the non-covalent dispersive interactions in the NCl_3_@C_2_N complex. The higher density of green patches was observed in the NBr_3_@C_2_N complex as compared to that of the NCl_3_@C_2_N complex.

The shattered spikes of the 2D-RGD graph of the NBr_3_@C_2_N complex also appeared at −0.01 a.u. of (*λ*_2_)*ρ* at the *X*-axis, which illustrated the stronger forces of interactions between the Br-atoms of NBr_3_ and the N & C-atoms of C_2_N as compared to that of NCl_3_@C_2_N complex (see Fig. 4). Among the NX_3_@C_2_N analytes, NI_3_ exhibited the strongest interaction with the C_2_N surface, which was confirmed by the thick and wide green patches seen in the 3D isosurface, and the projection of spikes between 0.01 to −0.02 a.u. at the *X*-axis of (*λ*_2_)*ρ*. The results of NCI analysis are consistent with those of the interaction energy of the NX_3_@C_2_N complexes.

### 3.3 SAPT0 analysis

**Fig. 5:**
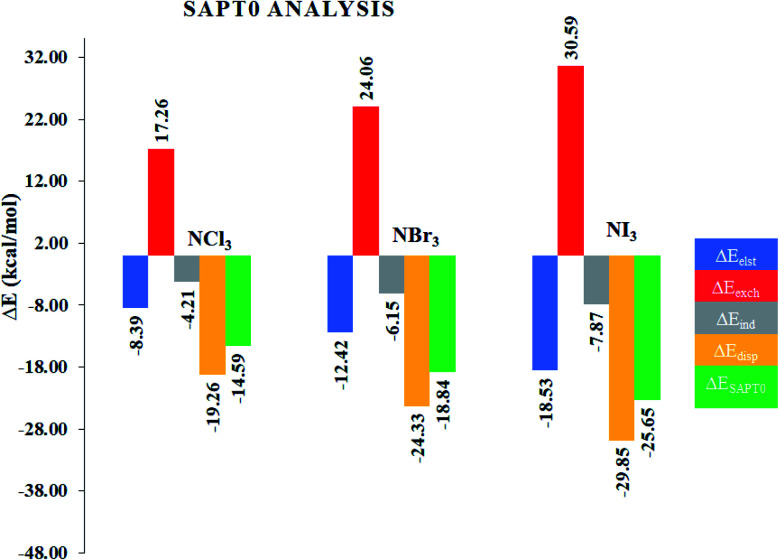
Graphical representation of the SAPT0 components of the NX_3_@C_2_N complexes.

Although NCI analysis indicates the presence of attractive and repulsive interactions between the components of a system, it would be better to understand the quantitative nature of the interactions in the studied complexes. In this regard, SAPT0 analysis was performed using the PSI4 software. The SAPT0 energy was divided into four terms, *i.e.*, *E*_disp_, *E*_exch_, *E*_elst_ and *E*_ind_. The results of the SAPT0 analysis are given in Table 2, whereas the graphical representation is given in Fig. 5. The SAPT0 values vary in each case, which indicates the different modes of interactions in the NX_3_@C_2_N complexes. The SAPT0 values observed for the NX_3_@C_2_N analytes were −14.59 kcal mol^−1^ (NCl_3_@C_2_N), −18.84 kcal mol^−1^ (NBr_3_@C_2_N) and −25.65 kcal mol^−1^ (NI_3_@C_2_N), respectively. The results of the SAPT0 energy components are consistent with the interaction energy results. The components of the SAPT0 energy for NCl_3_@C_2_N were −8.39 kcal mol^−1^ (*E*_elst_), −4.21 kcal mol^−1^ (*E*_ind_) and −19.26 kcal mol^−1^ (*E*_disp_). These results indicate that the dispersion (60.47%) was the major stabilizing factor, whereas electrostatic (26.33%), and induction (13.20%) were less prominent in the total interaction energy.

**Table 2:** SAPT0 energy component analysis of the NX_3_@C_2_N complexes

	Δ*E*_elst_ (%)	Δ*E*_exch_	Δ*E*_ind_ (%)	Δ*E*_disp_ (%)	Δ*E*_SAPT0_
NCl_3_@C_2_N	−8.39 (26.33)	17.26	−4.21 (13.20)	−19.26 (60.47)	−14.59
NBr_3_@C_2_N	−12.42 (28.95)	24.06	−6.15 (14.34)	−24.33 (56.70)	−18.84
NI_3_@C_2_N	−18.53 (32.94)	30.59	−7.87 (13.98)	−29.85 (53.08)	−25.65

In the case of the NBr_3_@C_2_N complex, the contributions of each component of energy towards total SAPT0 were −12.42 kcal mol^−1^ (*E*_elst_), −6.15 kcal mol^−1^ (*E*_ind_) and −24.33 kcal mol^−1^ (*E*_disp_). The trend of SAPT0 components (*E*_disp_ > *E*_elest_ > *E*_ind_) of NBr_3_@C_2_N is comparable to that of NCl_3_@C_2_N, where *E*_disp_ (56.70%) is dominant. The *E*_disp_ component in NBr_3_@C_2_N was less significant as compared to *E*_disp_ (60.47%) of the NCl_3_@C_2_N complex. Similarly, the increase in the *E*_ind_ (14.34%) and *E*_elst_ (28.95%) terms was noticed in the NBr_3_@C_2_N complex. Among the studied NX_3_@C_2_N complexes, the highest SAPT0 energy (−25.65 kcal mol^−1^) was observed for the NI_3_@C_2_N complex due to the stronger σ-hole interactions (halogen bonding) between the I-atoms of NI_3_ and the N-atoms of the C_2_N surface. The results (Table 2) revealed that the large contribution toward the total SAPT0 for the NI_3_@C_2_N complex was from *E*_disp_ (53.08%), quite similar to the NCl_3_@C_2_N and NBr_3_@C_2_N complexes. The contribution from *E*_elst_ (23.94%) and *E*_ind_ (13.98%) remained appreciable.

In the overall interactive components, the largest stabilizing factor remained *E*_disp_ (56.75%), whereas a moderate contribution was observed from *E*_elst_ (29.41%) and *E*_ind_ (14.34%), respectively. The trend of SAPT0 energy observed in the case of the NX_3_@C_2_N complexes is consistent with *E*_int_, and NCI analysis.

### 3.4 Quantum theory of atoms in molecules (QTAIM) theory

Quantum theory of the atoms in molecules (QTAIM) analysis is well-known because of its ability to describe various intra- and intermolecular interactions (H-bonding, ionic bonding, van der Waals interactions). Many topological parameters such as electron density (*ρ*), the Laplacian (∇^2^*ρ*), and total electron energy density (*H*(*r*)) are used at the bond critical point to quantify and understand the nature of the interactions.

The strength of a bond is measured by the electron density *ρ* value at BCPs, whereas the nature of interactions is characterized by the Laplacian (∇^2^*ρ*), potential energy density *V*(*r*), kinetic energy density *G*(*r*), and total electron energy density *H*(*r*). For shared shell interactions (such as covalent bonds), the electron density is greater than 0.1 a.u., while the Laplacian (∇^2^*ρ*) remains always large and negative. For closed interactions, (van der Waals, ionic and H-bonding), *ρ* lies in the range of 0.002–0.034 a.u. and the Laplacian (∇^2^*ρ*) is in the range of 0.024–0.139 a.u. Through the Espinosa approach of individual bond interaction energy, non-covalent interactions, particularly H-boding (3 to 10 kcal mol^−1^), can be better understood through eqn (5).^82–86^5
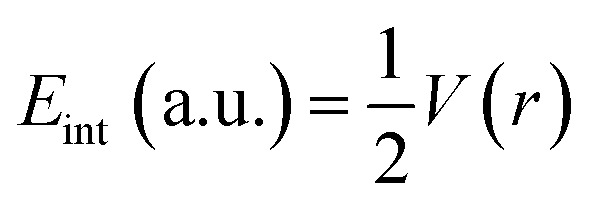


BCPs of closed and shared shells can also be explained by the following equations:6
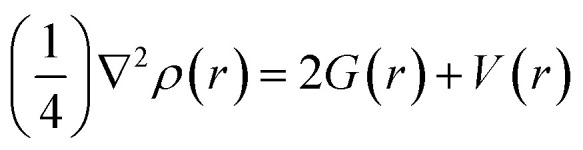

7 *H*(*r*) = *G*(*r*) + *V*(*r*)

**Fig. 6:**
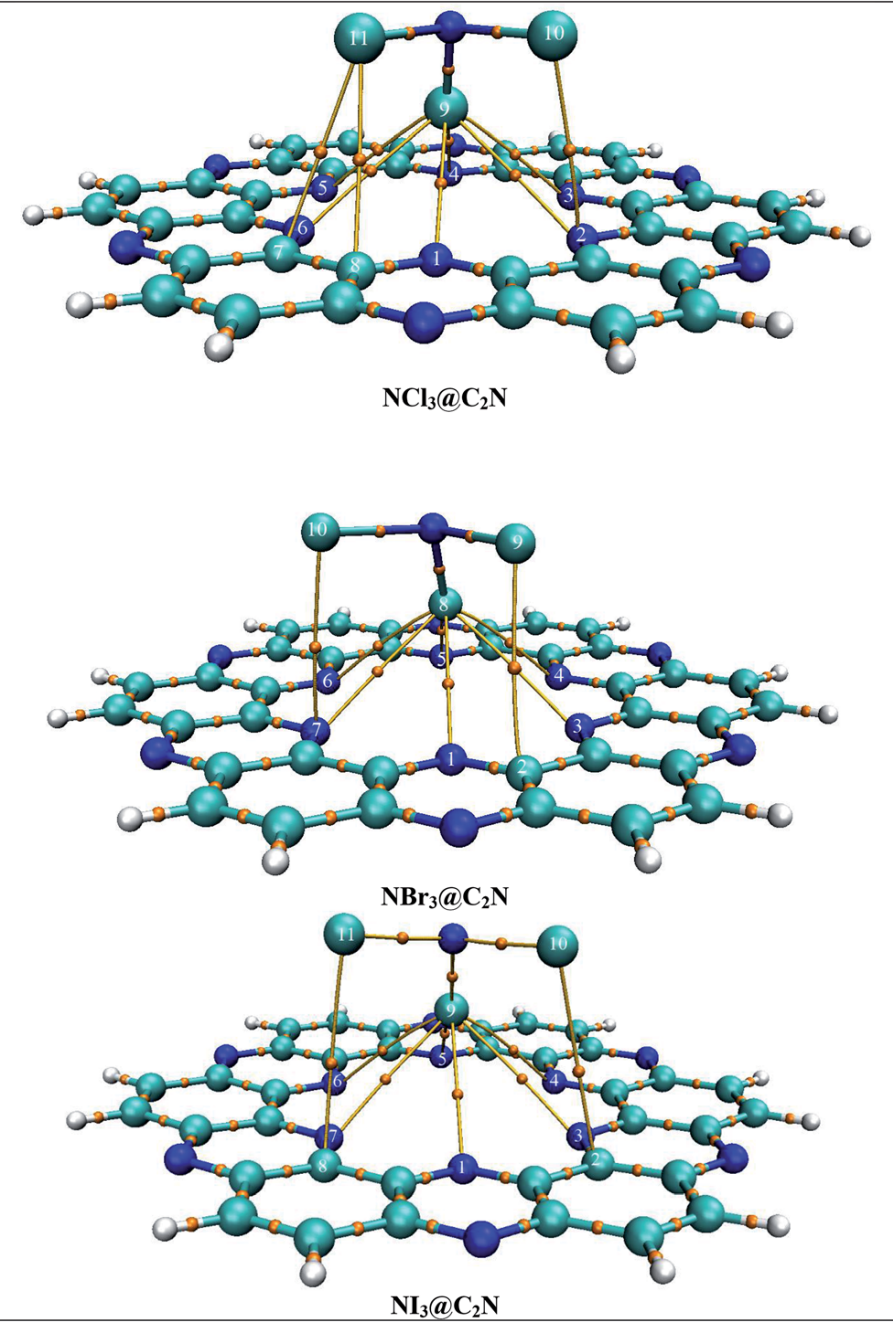
QTAIM topological analysis of NX_3_@C_2_N complexes for non-covalent interactions.

Here, *E*_int_, *V*(*r*), *G*(*r*) and *H*(*r*) are the individual bond interaction energy, potential energy density, kinetic energy density and total electron energy density at BCPs. *H*(*r*) < 0 indicates a shared shell, while *H*(*r*) > 0 reveals closed-shell interactions. We can also use the values of *H*(*r*) and ∇^2^*ρ*(*r*) to project the nature and type of interactions. The values of ∇^2^*ρ*(*r*) and *H*(*r*) greater than zero indicate non-covalent interactions, whereas ∇^2^*ρ*(*r*) and *H*(*r*) less than zero indicate covalent interactions, ∇^2^*ρ*(*r*) greater than zero and *H*(*r*) less than zero at BCPs indicate the existence of partial covalent character. The BCPs values of the closed-shell interactions of analytes@C_2_N complexes are given in Table S2 (ESI†) and topological complexes are shown in Fig. 6.

The observed numbers of BCPs in NX_3_@C_2_N complexes were 9 (NCl_3_@C_2_N), 8 (NBr_3_@C_2_N) and 8 (NI_3_@C_2_N) through QTAIM analysis. The evaluated *ρ*(*r*) and ∇^2^*ρ*(*r*) values of intermolecular BCPs were positive for all NX_3_@C_2_N complexes, which indicated the presence of non-covalent interactions (see Table S2†). In addition to this, the −*V*(*r*)/*G*(*r*) ratio was <1 and it ranged from 0.74 to 0.84, which confirmed the existence of weak non-covalent interactions. The value of *ρ*(*r*) and ∇^2^*ρ*(*r*) at BCPs also quantified the nature of the interactions. The *ρ*(*r*) of the various intermolecular interactions in the NX_3_@C_2_N complexes ranged from 0.005 to 0.013, while ∇^2^*ρ*(*r*) was from 0.009 to 0.057.

The observed *ρ*(*r*) and ∇^2^*ρ*(*r*) were the highest for the NI_3_@C_2_N complex due to the stronger halogen bonding between the I-atoms of NI_3_ and the N-atoms of C_2_N. The smallest values of *ρ*(*r*) and ∇^2^*ρ*(*r*) at BCPs were observed for the NCl_3_@C_2_N complex due to weak halogen bonding between the Cl-atoms of NCl_3_ and the N-atoms of C_2_N. The *ρ*(*r*) and ∇^2^*ρ*(*r*) values for the NBr_3_@C_2_N complex remained intermediate between NCl_3_@C_2_N and NI_3_@C_2_N. In addition to X–N (X = Cl, Br, I) interactions between the analyte and C_2_N, other weak interactions among the X-atoms of the analytes and the C-atoms of the C_2_N surface were also seen (see Fig. 6). The X-atoms of analytes pointing towards the surface acted as electrophiles and developed non-covalent interactions with the highly electronegative N-atoms. The rest of the X-atoms away from the surface acted as nucleophiles and produced weak van der Waals interactions with the C-atoms of the C_2_N surface (*vide infra*). Therefore, the trend established in the studied analytes@C_2_N complexes according to the topological parameters was NI_3_@C_2_N > NBr_3_@C_2_N and NCl_3_@C_2_N complexes.

The results of the individual bond interactions of the NX_3_@C_2_N complexes were between −0.59 and −3.19 kcal mol^−1^. The highest individual bond interactions were seen for the NI_3_@C_2_N complex. The other parameters (*H*(*r*), −*V*(*r*)/*G*(*r*)) were also in accordance with the *ρ*(*r*) and ∇^2^*ρ*(*r*) of the studied BCPs. This was due to the low individual bond interaction energy and longer interaction distance (1.8 Å) between interacting systems. The trend of individual bond interactions was also comparable to the rest of the topological parameters and consistent with the interaction energy results, NCI and SAPT0 analysis.

## 4. Electronic properties

### 4.1 Electron density differences analysis

**Fig. 7:**
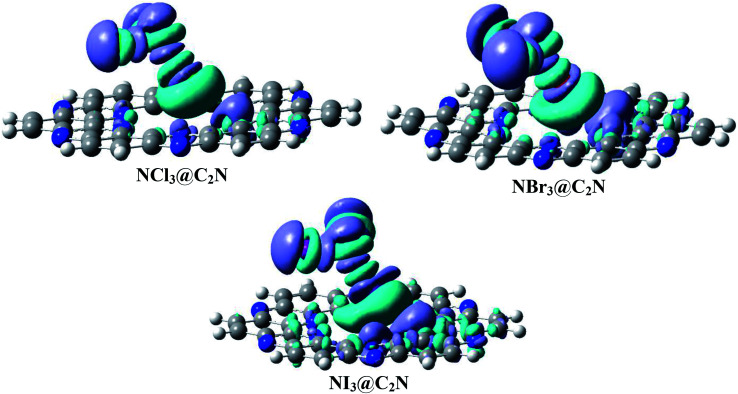
Electron density difference plots of analytes@C_2_N (iso value = 0.004 a.u.). Purple represents the accumulation of electron density, while green surfaces show the depletion of electron density.

We calculated the three-dimensional electron density differences and evaluated the charge transfer between analytes (NX_3_) and the C_2_N surface. The isosurfaces obtained by electron density differences are given in Fig. 7. The isosurface of NX_3_@C_2_N consists of green and purple shades to represent the depletion and accumulation of charge densities, respectively. It is worth mentioning that the accumulation of charge density takes place where the atoms of analytes interact with the C_2_N surface of C_2_N (see Fig. 7), this shows the existence of van der Waals interactions between the atoms of NX_3_ and the C_2_N surface.

The electron density differences (Fig. 7) clearly indicate that the flow of electron was from the C_2_N surface to analytes, as revealed by the appearance of green surfaces on the X-atoms (Cl, Br and I) of analytes and the purple surfaces on the N-atoms of C_2_N. The charge transfer from C_2_N to the X-atoms of analytes is due to halogen bonding. The EDD results in Fig. 7 also indicate that the X-atom of NX_3_ oriented toward the C_2_N surface shows the electrophilic character (*σ*-hole) due to the formation of green surfaces. The X-atoms of analytes projected away from the C_2_N surface show nucleophilic character due to the formation of purple surfaces. The maximum accumulation of densities was seen between the Br-atoms of NBr_3_ and the N-atoms of C_2_N, which is consistent with the results of NBO analysis.

### 4.2 Natural bond orbital analysis

Charge analysis provides valuable information about the amount of charge transfer between the studied systems. During the interaction of NX_3_ with the C_2_N surface, the analytes take the negative charge and deliver positive charge to the C_2_N surface (Table 3). The charges transferred from the C_2_N surface to the analytes were −0.001*e*^−^ (NCl_3_), −0.006*e*^−^ (NBr_3_) and −0.004*e*^−^ (NI_3_). Surprisingly, the charge analysis results show that NBr_3_ extracted a relatively higher amount of charge (−0.006*e*^−^) from the C_2_N surface as compared to NCl_3_ (−0.001*e*^−^) and NI_3_ (−0.004*e*^−^). This finding of charge analysis in the case of the NBr_3_@C_2_N complex is somewhat contradictory to the interaction energy results. In the case of the NI_3_@C_2_N complex, the higher interaction energy resulted from the strong halogen bonding of highly polarizable I-atoms of NI_3_ with the N and C atoms of the C_2_N surface (*vide supra*). The charge extracted by NI_3_ from C_2_N was slightly lower than NBr_3_ due to the greater interaction distance in the former (from the C_2_N surface). The lowest charge transfer was observed in the case of NCl_3_ (−0.001*e*^−^) due to its weak halogen bonding. The halogen bonding increased in the order of F < Cl < Br < I due to σ-hole character. The NBO charge analysis values of NCl_3_ were consistent with their low interaction energy results.

**Table 3:** The results of the frontier molecular orbital and NBO analyses of the analytes@C_2_N surface

Analytes	HOMO (eV)	LUMO (eV)	*E*_H–L_ gap (eV)	NBO (*e*^−^) on analytes
C_2_N	−8.06	−2.35	5.71	—
NCl_3_@C_2_N	−8.12	−2.54	5.58	−0.001
NBr_3_@C_2_N	−7.98	−2.69	5.29	−0.006
NI_3_@C_2_N	−6.86	−2.71	4.15	−0.004

### 4.3 Frontier molecular orbital (FMO) analysis

**Fig. 8:**
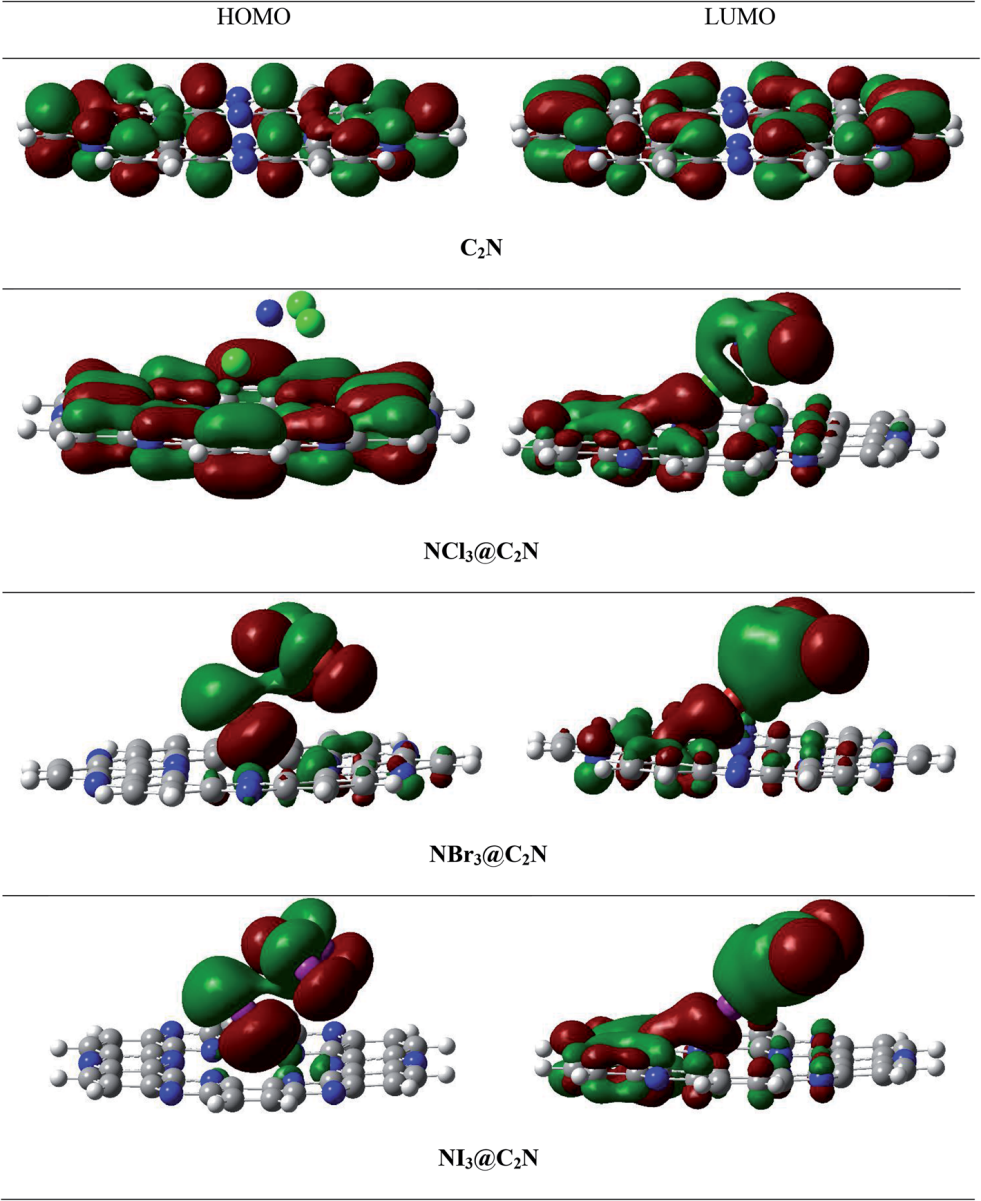
Top and side views of the HOMO and LUMO densities of the analytes@C_2_N surface.

To better understand electronic changes caused by the interaction of analytes on the C_2_N surface, FMOs were analyzed before and after the complexation of analytes (NX_3_) over the C_2_N surface. Energies of the highest occupied molecular orbitals (*E*_HOMO_), lowest unoccupied molecular orbital (*E*_LUMO_) and *E*_H–L_ gap for NX_3_@C_2_N complexes are given in Table 3, and their orbital densities are given in Fig. 8. The *E*_HOMO_ and *E*_LUMO_ of the bare C_2_N surface were −8.06 eV and −2.35 eV, which resulted in a 5.71 eV *E*_H–L_ gap. The orbital density in the HOMO was distributed on the entire C_2_N surface, while the density of LUMO was located mostly on the nitrogen atoms of the C_2_N surface.

The interaction of analytes (NX_3_) over the C_2_N surface not only causes changes in the *E*_HOMO_ and *E*_LUMO_ but also in their orbital densities. The *E*_H–L_ gaps observed upon the interaction of the analytes@C_2_N surfaces were 5.58 eV (NCl_3_@C_2_N), 5.29 eV (NBr_3_@C_2_N) and 4.15 eV (NI_3_@C_2_N). For NCl_3_@C_2_N, *E*_HOMO_ and *E*_LUMO_ were −8.12 eV and −2.54 eV, respectively, which showed that the energies of both HOMO and LUMO were affected by complexation. The energies of the HOMO and LUMO slightly decreased with the adsorption of NCl_3_ on the C_2_N surface. However, the effect was more pronounced on the LUMO, which resulted in the decrease in the *E*_H–L_ gap to 5.58 eV.

A significant decrease in the *E*_H–L_ gap (5.29 eV) was observed in the NBr_3_@C_2_N complex. Table 3 shows that the adsorption of NBr_3_ on C_2_N caused the energy of the HOMO to increase from (−8.06 eV to −7.98 eV) but the energy of the LUMO decreased from −2.35 to −2.69 eV), which resulted in a relatively small *E*_H–L_ gap (5.29 eV). Among the studied NX_3_@C_2_N complexes, the highest decrease in the *E*_H–L_ gap (4.15 eV) was observed for the NI_3_@C_2_N complex. The substantial increase in the energy of the HOMO (−6.86 eV) and the decrease in the energy of the LUMO (−2.71 eV) resulted in the lowering of the *E*_H–L_ gap. The results of the FMO analysis revealed that the complexation of NI_3_ over the C_2_N surface increased the conductivity of C_2_N due to the highest decreases in the *E*_H–L_ gap of C_2_N (4.15 eV) as compared to other analytes. These results also showed the strong sensitivity and selectivity of the C_2_N surface towards NI_3_ as compared to the rest of the analytes. FMO analysis revealed that the adsorption of analytes produced a more pronounced effect on the HOMO energies as compared to the LUMO, which led to the greatest decrease in the *E*_H–L_ gap.

**Fig. 9:**
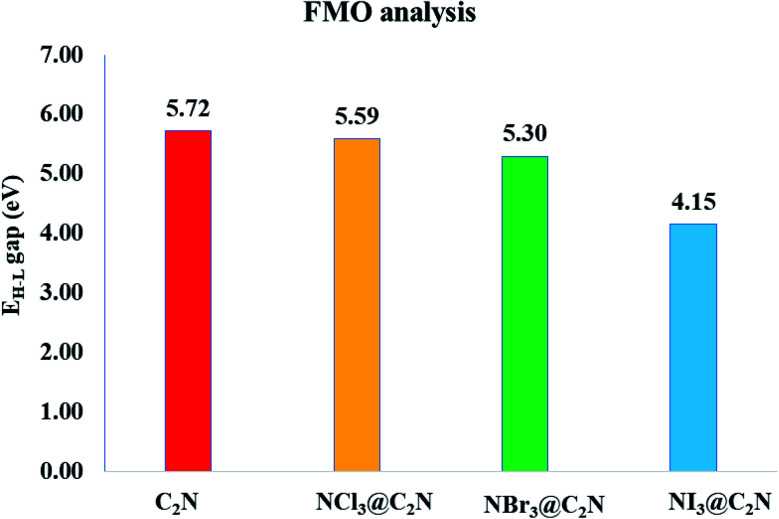
Graphical representation of the *E*_H–L_ gap of the bare C_2_N surface and the NX_3_@C_2_N complexes.

The orbital plots showed significant changes in the HOMO and LUMO densities with the adsorption of analytes over the C_2_N surface. The HOMO–LUMO density analysis of the NCl_3_@C_2_N complex showed that the HOMO density was completely concentrated over the C_2_N surface, while the LUMO density was distributed on NCl_3_ as well as on half of the C_2_N. In the case of the NBr_3_@C_2_N complex, the distribution pattern of the HOMO densities was different from the NCl_3_@C_2_N complex, however, the LUMO distribution pattern was similar to the NCl_3_@C_2_N complex. The HOMO density was mainly contributed by NBr_3_ with a small contribution from the N-atoms of the C_2_N surface. In the NI_3_@C_2_N complex, the pattern of orbital density distribution is comparable to the NBr_3_@C_2_N complex, where the HOMO density is located on the analytes, whereas LUMO was distributed between the analytes and the C_2_N surface (see Fig. 8). The *E*_H–L_ gap is presented in graphical form in Fig. 9.

FMO analysis revealed that the decrease in the *E*_H–L_ gap was observed when the electronic transition occurred from the analyte to the C_2_N surface, which was evident in the case of the NI_3_@C_2_N complex. These findings clearly indicate that the shift in the electronic density is very important for the electrochemical identification of analytes over the C_2_N surface. These results indicate that the C_2_N sensor response is more toward NI_3_ as compared to the rest of the studied analytes. The FMO results are in strong agreement with the NCI and SAPT0, QTAIM and interaction energy results.

## 5. Conclusion

The adsorption of the NX_3_ analytes (NCl_3_, NBr_3_ and NI_3_) over the C_2_N surface was investigated by using DFT calculations at the M05-2X/LANL2DZ level of theory. For a thorough understanding of the NX_3_@C_2_N complexes, adsorption energy, NCI, QTAIM, SAPT0, NBO, EDD and FMO analyses were performed. The interaction energies of the NX_3_@C_2_N complexes were in the range of −10.85 to −16.31 kcal mol^−1^. These results demonstrate that the physisorption phenomena were represented for the adsorption of analytes on the C_2_N surface. The interaction energy trends observed for NX_3_@C_2_N complexes are NCl_3_@C_2_N > NBr_3_@C_2_N > NI_3_@C_2_N, respectively. The stable geometries of the analytes@C_2_N complexes were mainly obtained by the interaction of the X-atoms (Cl, Br, I) of the analytes with the C_2_N surface.

The 3D isosurfaces and 2D-RGD graph of the NCI analysis also confirmed the existence of halogen bonding interactions among the studied systems. The halogen bonding was also quantified by SAPT0 component energy analysis. The SAPT0 results revealed that dispersion was the most prominent contributor to SAPT0 energy, whereas lower contributions were observed from Δ*E*_elst_ (29.41%) and Δ*E*_ind_ (14.34%). The topological parameters ((*ρ*(*r*) and ∇^2^*ρ*(*r*)) of QTAIM analysis also confirmed the presence of halogen bonding between the X-atoms of NX_3_ and C_2_N. NBO analysis showed that in all cases, the transfer of charge happened from C_2_N to the analytes. A significant amount of charge was received by NBr_3_ (−0.006*e*^−^) and NI_3_ (0.004*e*^−^). EDD analysis also validated the NCI, QTAIM and NBO analyses. FMO analysis revealed that the adsorption of NI_3_ on the C_2_N surface caused significant changes in the *E*_HOMO–LUMO_ gap (from 5.71 to 4.15 eV) as compared to bare C_2_N units, which showed the strong sensitivity and selectivity of the C_2_N surface towards NI_3_ as compared to the rest of the analytes. The complexes of NCl_3_@C_2_N (5.58 eV) and NBr_3_@C_2_N (5.25 eV) failed to bring significant changes in the *E*_HOMO–LUMO_ energy gaps and were less selective by the C_2_N surface. It is worth mentioning that in all complexes, a significant difference in the *E*_HOMO–LUMO_ gap was seen when charge transfer happened from the analyte to the C_2_N surface.

## Conflicts of interest

The authors declare that they have no known competing financial interests or personal relationships that could have appeared to influence the work reported in this paper.

## Supplementary Material

RA-010-D0RA04930A-s001

## References

[cit1] Matyáš R. and Pachman J., Nitrogen Halides, in Prim. Explos., Springer Berlin Heidelberg, Berlin, Heidelberg, 2013, pp. 289–302, 10.1007/978-3-642-28436-6_11

[cit2] Tornieporth-Oetting I., Klapötke T. (1990). Nitrogen Triiodide. Angew. Chem., Int. Ed. Engl..

[cit3] Noyes W. A. (1928). The interaction between nitrogen trichloride and nitric oxide. reactions of compounds with odd electrons 1. J. Am. Chem. Soc..

[cit4] Galal-Gorchev H., Morris J. C. (1965). Formation and Stability of Bromamide, Bromimide, and Nitrogen Tribromide in Aqueous Solution. Inorg. Chem..

[cit5] Maka K., Norval G. W. (2013). Optimising chlorine compression design with vapour phase nitrogen trichloride destruction. Can. J. Chem. Eng..

[cit6] Gérardin F., Cloteaux A., Guillemot M., Faure M., André J. C. (2013). Photocatalytic Conversion of Gaseous Nitrogen Trichloride into Available Chlorine—Experimental and Modeling Study. Environ. Sci. Technol..

[cit7] Okada K., Akiyoshi M., Ishizaki K., Sato H., Matsunaga T. (2014). Analysis of an explosion accident of nitrogen trichloride in a waste liquid containing ammonium ion and platinum black. J. Hazard. Mater..

[cit8] Nguyen T.-H., Chevallier E., Garcia J., Nguyen T.-D., Laurent A.-M., Beaubestre C., Karpe P., Tran-Thi T.-H. (2013). Innovative colorimetric sensors for the detection of nitrogen trichloride at ppb level in swimming pools. Sens. Actuators, B.

[cit9] Ganji M. D., Tajbakhsh M., Laffafchy M. (2010). Nerve agents interacting with single wall carbon nanotubes: Density functional calculations. Solid State Sci..

[cit10] Horrillo M. C., Martí J., Matatagui D., Santos J. P., Sayago I., Gutiérrez J., Martin-Fernandez I., Ivanov P., Gràcia I., Cané C. (2011). Single-walled carbon nanotube microsensors for nerve agent simulant detection. Sens. Actuators, B.

[cit11] Sherma J., Zweig G. (1985). Pesticides. Anal. Chem..

[cit12] Ji X., Zheng J., Xu J., Rastogi V. K., Cheng T.-C., DeFrank J. J., Leblanc R. M. (2005). (CdSe)ZnS Quantum Dots and Organophosphorus Hydrolase Bioconjugate as Biosensors for Detection of Paraoxon. J. Phys. Chem. B.

[cit13] Potyrailo R. A. (2006). Polymeric Sensor Materials: Toward an Alliance of Combinatorial and Rational Design Tools. Angew. Chem., Int. Ed..

[cit14] Korotcenkov G. (2007). Metal oxides for solid-state gas sensors: what determines our choice. Mater. Sci. Eng., B.

[cit15] Sahoo H. R., Kralj J. G., Jensen K. F. (2007). Multistep Continuous-Flow Microchemical Synthesis Involving Multiple Reactions and Separations. Angew. Chem., Int. Ed..

[cit16] Lu G., Hupp J. T. (2010). Metal–Organic Frameworks as Sensors: A ZIF-8 Based Fabry–Pérot Device as a Selective Sensor for Chemical Vapors and Gases. J. Am. Chem. Soc..

[cit17] Zhou X., Su Z., Chen H., Xiao X., Qin Y., Yang L., Yan Z., Sun W. (2018). Capture of pure toxic gases through porous materials from molecular simulations. Mol. Phys..

[cit18] Li J.-R., Kuppler R. J., Zhou H.-C. (2009). Selective gas adsorption and separation in metal–organic frameworks. Chem. Soc. Rev..

[cit19] Yuan J., Li G., Yang B., Zhang J., Li Z., Chen H. (2016). Selective adsorption of ethylene on bimetallic CuVn+/0 (*n* = 1-5) clusters: a theoretical study. Comput. Mater. Sci..

[cit20] Online V. A. (2016). Long range corrected-wPBE based analysis of the H_2_O adsorption on magnetic BC 3 nanosheets. RSC Adv..

[cit21] Sangiovanni D. G., Gueorguiev G. K., Kakanakova-Georgieva A. (2018). *Ab initio* molecular dynamics of atomic-scale surface reactions: Insights into metal organic chemical vapor deposition of AlN on graphene. Phys. Chem. Chem. Phys..

[cit22] Anota E. C., Juárez A. R., Castro M., Cocoletzi H. H. (2013). A density functional theory analysis for the adsorption of the amine group on graphene and boron nitride nanosheets. J. Mol. Model..

[cit23] Limon P., Miralrio A., Castro M. (2018). Adsorption and dissociation of carbon monoxide on iron and iron-carbon clusters: Fen + 2CO and FenC + 2CO, *n* = 4 and 7. A theoretical study. Comput. Theor. Chem..

[cit24] Baughman R. H. (2002). Carbon Nanotubes–the Route Toward Applications. Science.

[cit25] Broitman E., Gueorguiev G. K., Furlan A., Son N. T., Gellman A. J., Stafström S., Hultman L. (2008). Water adsorption on fullerene-like carbon nitride overcoats. Thin Solid Films.

[cit26] Deng Z.-Y., Zhang J.-M., Xu K.-W. (2016). Adsorption of SO_2_ molecule on doped (8, 0) boron nitride nanotube: a first-principles study. Physica E Low Dimens. Syst. Nanostruct..

[cit27] Rostami Z., Soleymanabadi H. (2017). Investigation of phosgene adsorption behavior on aluminum nitride nanocones: density functional study. J. Mol. Liq..

[cit28] Broitman E., Furlan A., Gueorguiev G. K., Czigány Z., Tarditi A. M., Gellman A. J., Stafström S., Hultman L. (2009). Water adsorption on phosphorous-carbide thin films. Surf. Coat. Technol..

[cit29] Goyenola C., Stafström S., Schmidt S., Hultman L., Gueorguiev G. K. (2014). Carbon Fluoride, CF_x_: Structural Diversity as Predicted by First Principles. J. Phys. Chem. C.

[cit30] dos Santos R. B., Rivelino R., Mota F. de B., Gueorguiev G. K. (2012). Exploring Hydrogenation and Fluorination in Curved 2D Carbon Systems: A Density Functional Theory Study on Corannulene. J. Phys. Chem. A.

[cit31] Mahmood J., Lee E. K., Jung M., Shin D., Jeon I.-Y., Jung S.-M., Choi H.-J., Seo J.-M., Bae S.-Y., Sohn S.-D., Park N., Oh J. H., Shin H.-J., Baek J.-B. (2015). Nitrogenated holey two-dimensional structures. Nat. Commun..

[cit32] Hussain T., Searles D. J., Hankel M. (2020). Insights into the trapping mechanism of light metals on C2N-h2D: utilisation as an anode material for metal ion batteries. Carbon.

[cit33] Xu J., Mahmood J., Dou Y., Dou S., Li F., Dai L., Baek J.-B. (2017). 2D Frameworks of C_2_N and C_3_N as New Anode Materials for Lithium-Ion Batteries. Adv. Mater..

[cit34] Zhang Q., Wang Y., Seh Z. W., Fu Z., Zhang R., Cui Y. (2015). Understanding the Anchoring Effect of Two-Dimensional Layered Materials for Lithium–Sulfur Batteries. Nano Lett..

[cit35] Guan Z., Lian C.-S., Hu S., Ni S., Li J., Duan W. (2017). Tunable Structural, Electronic Properties of Layered Two-Dimensional C_2_N and MoS_2_ van der Waals Heterostructure as Photovoltaic Material. J. Phys. Chem. C.

[cit36] Li X., Zhong W., Cui P., Li J., Jiang J. (2016). Design of Efficient Catalysts with Double Transition Metal Atoms on C_2_N Layer. J. Phys. Chem. Lett..

[cit37] Tsoeu S. E., Opoku F., Govender P. P. (2020). Tuning the electronic, optical and structural properties of GaS/C_2_N van der Waals heterostructure for photovoltaic application: first-principle calculations. SN Appl. Sci..

[cit38] Bhattacharyya K., Pratik S. M., Datta A. (2018). Controlled Pore Sizes in Monolayer C N act as Ultrasensitive Probes for Detection of Gaseous Pollutants ( HF, HCN and H S) Controlled Pore Sizes in Monolayer C_2_N act as Ultrasensitive Probes for Detection of Gaseous Pollutants (HF, HCN and H_2_S)K. J. Phys. Chem. C.

[cit39] Su Y., Ao Z., Ji Y., Li G., An T. (2018). Adsorption mechanisms of different volatile organic compounds onto pristine C_2_N and Al-doped C_2_N monolayer: a DFT investigation. Appl. Surf. Sci..

[cit40] Li C., Xu Y., Sheng W., Yin W.-J., Nie G.-Z., Ao Z. (2020). A promising blue phosphorene/C_2_N van der Waals type-II heterojunction as a solar photocatalyst: a first-principles study. Phys. Chem. Chem. Phys..

[cit41] Xu B., Xiang H., Wei Q., Liu J. Q., Xia Y. D., Yin J., Liu Z. G. (2015). Two-dimensional graphene-like C_2_N: an experimentally available porous membrane for hydrogen purification. Phys. Chem. Chem. Phys..

[cit42] Yar M., Hashmi M. A., Ayub K. (2019). Nitrogenated holey graphene (C_2_N) surface as highly selective electrochemical sensor for ammonia. J. Mol. Liq..

[cit43] Bhattacharyya K., Pratik S. M., Datta A. (2018). Controlled Pore Sizes in Monolayer C_2_N Act as Ultrasensitive Probes for Detection of Gaseous Pollutants (HF, HCN, and H_2_S). J. Phys. Chem. C.

[cit44] Yar M., Ayub K. (2020). Expanding the horizons of covalent organic frameworks to electrochemical sensors; a case study of CTF-FUM. Microporous Mesoporous Mater..

[cit45] Sajid H., Ayub K., Mahmood T. (2020). Exceptionally high NLO response and deep ultraviolet transparency of superalkali doped macrocyclic oligofuran rings. New J. Chem..

[cit46] Wasim F., Mahmood T., Ayub K. (2016). An accurate cost effective DFT approach to study the sensing behaviour of polypyrrole towards nitrate ions in gas and aqueous phases. Phys. Chem. Chem. Phys..

[cit47] Sajid H., Mahmood T., Ayub K. (2017). An accurate comparative theoretical study of the interaction of furan, pyrrole, and thiophene with various gaseous analytes. J. Mol. Model..

[cit48] Ayub K. (2017). Transportation of hydrogen atom and molecule through X12Y12 nano-cages. Int. J. Hydrogen Energy.

[cit49] Munsif S., Ayub K. (2018). Permeability and storage ability of inorganic X12Y12 fullerenes for lithium atom and ion. Chem. Phys. Lett..

[cit50] Sajid H., Ayub K., Arshad M., Mahmood T. (2019). Highly selective acridinium based cyanine dyes for the detection of DNA base pairs (adenine, cytosine, guanine and thymine). Comput. Theor. Chem..

[cit51] Sajid H., Mahmood T., Ayub K. (2018). High sensitivity of polypyrrole sensor for uric acid over urea, acetamide and sulfonamide: a density functional theory study. Synth. Met..

[cit52] Sajid H., Mahmood T., Ayub K. (2018). High sensitivity of polypyrrole sensor for uric acid over urea, acetamide and sulfonamide: a density functional theory study. Synth. Met..

[cit53] He G., He H. (2016). DFT studies on the heterogeneous oxidation of SO_2_ by oxygen functional groups on graphene. Phys. Chem. Chem. Phys..

[cit54] Goyenola C., Stafström S., Hultman L., Gueorguiev G. K. (2012). Structural Patterns Arising during Synthetic Growth of Fullerene-Like Sulfocarbide. J. Phys. Chem. C.

[cit55] Pacheco J. M., Gueorguiev G. K., Martins J. L. (2002). First-principles study of the possibility of condensed phases of endohedral silicon cage clusters. Phys. Rev. B: Condens. Matter Mater. Phys..

[cit56] Sajid H., Mahmood T., Mahmood M. H. R., Ayub K. (2019). Comparative investigation of sensor application of polypyrrole for gaseous analytes. J. Phys. Org. Chem..

[cit57] Sajid H., Mahmood T., Ayub K. (2018). High sensitivity of polypyrrole sensor for uric acid over urea, acetamide and sulfonamide: a density functional theory study. Synth. Met..

[cit58] Sajid H., Mahmood T., Mahmood M. H. R., Ayub K. (2019). Comparative investigation of sensor application of polypyrrole for gaseous analytes. J. Phys. Org. Chem..

[cit59] Gaussview I. , Viewing Gaussian Structures with GaussView, pp. 1–25

[cit60] Sajjad S., Maria, Mahmood T., Ayub K. (2017). Benchmark study of structural and vibrational properties of scandium clusters. J. Mol. Struct..

[cit61] Yar M. , Hashmi M. A., Khan A. and Ayub K., Carbon nitride 2-D surface as a highly selective electrochemical sensor for V-series nerve agents, Elsevier B.V., 2020, 10.1016/j.molliq.2020.113357

[cit62] Lu T., Chen F. (2012). Multiwfn: a multifunctional wavefunction analyzer. J. Comput. Chem..

[cit63] Cukrowski I., De Lange J. H., Adeyinka A. S., Mangondo P. (2015). Evaluating common QTAIM and NCI interpretations of the electron density concentration through IQA interaction energies and 1D cross-sections of the electron and deformation density distributions. Comput. Theor. Chem..

[cit64] Trendafilova N., Bauer G., Mihaylov T. (2004). DFT and AIM studies of intramolecular hydrogen bonds in dicoumarols. Chem. Phys..

[cit65] Ghogomu J. N., Nkungli N. K. (2017). DFT Studies and Topological Analyses of Electron Density on Acetophenone and Propiophenone Thiosemicarbazone Derivatives as Covalent Inhibitors of Falcipain-2, a Major Plasmodium Falciparum Cysteine Protease. Phys. Chem. Res..

[cit66] Bhadane S. A., Lande D. N., Gejji S. P. (2016). Understanding Binding of Cyano-Adamantyl Derivatives to Pillar[6]arene Macrocycle from Density Functional Theory. J. Phys. Chem. A.

[cit67] Marana N. L., Casassa S. M., Sambrano J. R. (2017). Adsorption of NH_3_ with Different Coverages on Single-Walled ZnO Nanotube: DFT and QTAIM Study. J. Phys. Chem. C.

[cit68] Venkataramanan N. S., Suvitha A., Kawazoe Y. (2018). Unravelling the nature of binding of cubane and substituted cubanes within cucurbiturils: a DFT and NCI study. J. Mol. Liq..

[cit69] Wang Z., Chen M., Huang Y., Shi X., Zhang Y., Huang T. (2018). Self-Assembly Synthesis of Boron-Doped Graphitic Carbon Nitride Hollow Tubes for Enhanced Photocatalytic NO_x_ Removal under Visible Light Applied Catalysis B : Environmental Self-Assembly Synthesis of Boron-Doped Graphitic Carbon Nitride Hollow Tubes for e. Appl. Catal. B Environ..

[cit70] Hussain M., Song X., Shah S., Hao C. (2019). Spectrochim. Acta, Part A.

[cit71] Esra M. D., Nejadebrahimi B. (2019). Applied Surface Science Theoretical insights into hydrogenation of CO_2_ to formic acid over a single Co atom incorporated nitrogen-doped graphene, a DFT study. Appl. Surf. Sci..

[cit72] Sarr S., Graton J., Montavon G., Pilmé J., Galland N. (2020). On the Interplay between Charge-Shift Bonding and Halogen Bonding. ChemPhysChem.

[cit73] Metrangolo P., Meyer F., Pilati T., Resnati G., Terraneo G. (2008). Halogen Bonding in Supramolecular Chemistry. Angew. Chem,. Int. Ed..

[cit74] Ayub K., Mahmood T. (2013). DFT studies of halogen bonding abilities of nitrobenzene with halogens and chlorofluorocarbons. J. Chem. Soc. Pak..

[cit75] Meyer F., Dubois P. (2013). Halogen bonding at work: recent applications in synthetic chemistry and materials science. CrystEngComm.

[cit76] Mukherjee A., Tothadi S., Desiraju G. R. (2014). Halogen Bonds in Crystal Engineering: Like Hydrogen Bonds yet Different. Acc. Chem. Res..

[cit77] Han Z., Czap G., Chiang C., Xu C., Wagner P. J., Wei X., Zhang Y., Wu R., Ho W. (2017). Imaging the halogen bond in self-assembled halogenbenzenes on silver. Science.

[cit78] Margiotta E., van der Lubbe S. C. C., de Azevedo Santos L., Paragi G., Moro S., Bickelhaupt F. M., Fonseca Guerra C. (2020). Halogen Bonds in Ligand–Protein Systems: Molecular Orbital Theory for Drug Design. J. Chem. Inf. Model..

[cit79] Sutar R. L., Engelage E., Stoll R., Huber S. M. (2020). Bidentate chiral bis(imidazolium)-based halogen bond donors: synthesis and first applications in enantioselective recognition and catalysis. Angew. Chem., Int. Ed..

[cit80] Politzer P., Murray J. S., Clark T. (2013). Halogen bonding and other σ-hole interactions: a perspective. Phys. Chem. Chem. Phys..

[cit81] Ford M. C., Ho P. S. (2016). Computational Tools to Model Halogen Bonds in Medicinal Chemistry. J. Med. Chem..

[cit82] Su H., Wu Q., Wang H., Wang H. (2018). Ab initio calculations, structure, NBO and NCI analyses of X–H⋯π interactions. Chem. Phys. Lett..

[cit83] Fedorov A. Y., Drebushchak T. N., Tantardini C. (2019). Seeking the best model for non-covalent interactions within the crystal structure of meloxicam. Comput. Theor. Chem..

[cit84] Benaissi H., Drissi M., Yahiaoui S., Megrouss Y., Chouaih A., Hamzaoui F. (2018). Hirshfeld surface analysis, topological features and nonlinear optical properties of phthalonitrile derivative , low temperature experimental charge density and quantum chemistry studies. J. Optoelectron. Biomed. Mater..

[cit85] Espinosa E., Molins E., Lecomte C. (1998). Hydrogen bond strengths revealed by topological analyses of experimentally observed electron densities. Chem. Phys. Lett..

[cit86] Steiner T. (2002). The Hydrogen Bond in the Solid State. Angew. Chem., Int. Ed..

