# Supplementary material for: The C2N surface as a highly selective sensor for the detection of nitrogen iodide from a mixture of NX3 (X = Cl, Br, I) explosives
Source: RSC Adv. 2020 Aug 27;10(53):31997–2010. doi: 10.1039/d0ra04930a (PMC9056556; doi:10.1039/d0ra04930a)
Supplement: RA-010-D0RA04930A-s001 [file RA-010-D0RA04930A-s001.pdf]

Electronic Supplementary Material (ESI) for RSC Advances.
This journal is © The Royal Society of Chemistry 2020

Supporting Information

C_2N surface as high selective sensor for the detection of nitrogen iodide from a mixture of NX_3 (X= Cl, Br, I) explosives

Muhammad Yar[a], *Muhammad Ali Hashmi*[b] *and Khurshid Ayub*[a,] *

[a]Department of Chemistry, COMSATS University, Abbottabad Campus, KPK, Pakistan 22060

[b]Department of Chemistry, University of Education, Attock Campus, Attock, Punjab, Pakistan 43600

*Corresponding author. Tel: +92-992-383591. E-mail: khurshid@cuiatd.edu.pk (Khurshid Ayub)

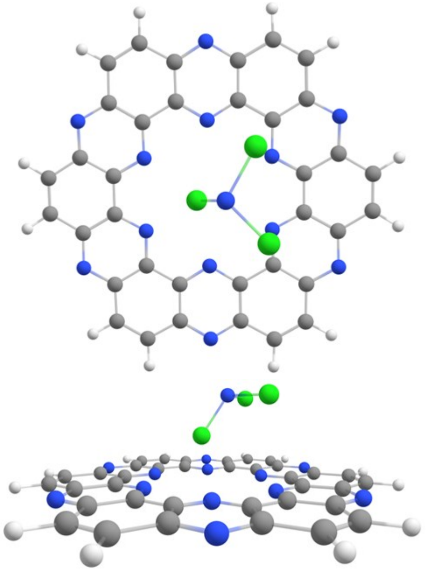

A

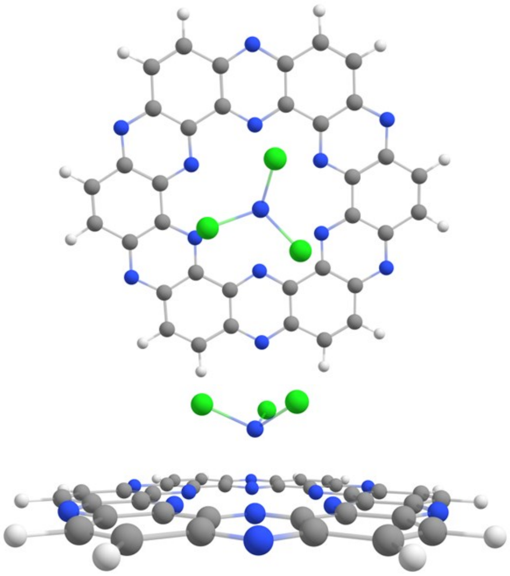

B

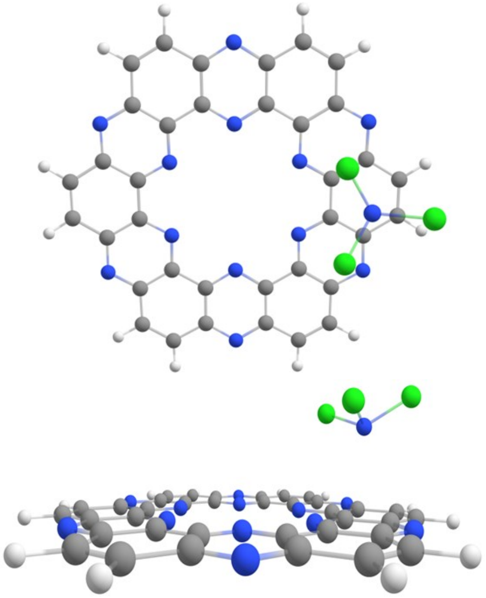

C

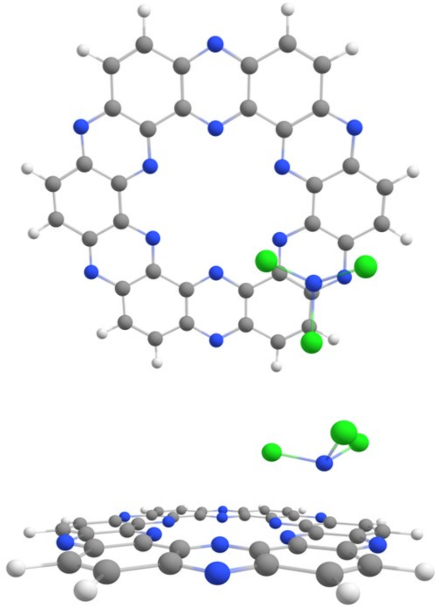

D

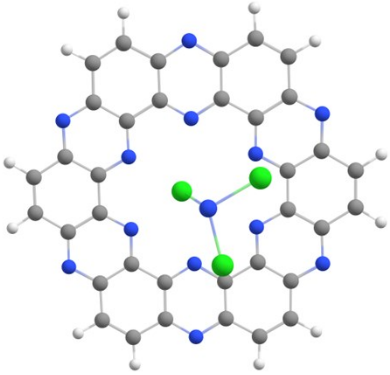

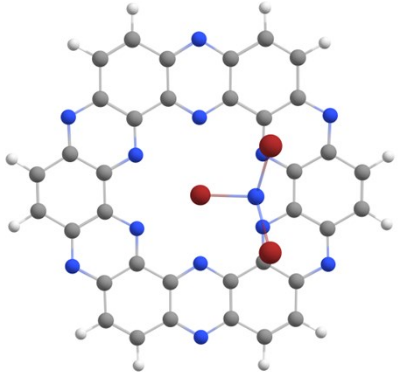

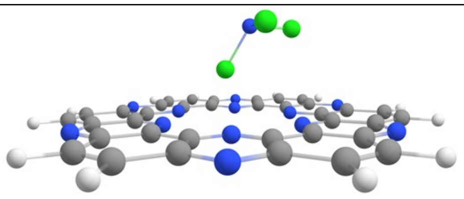

E

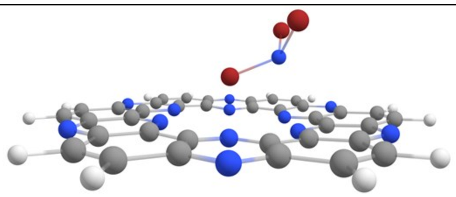

F

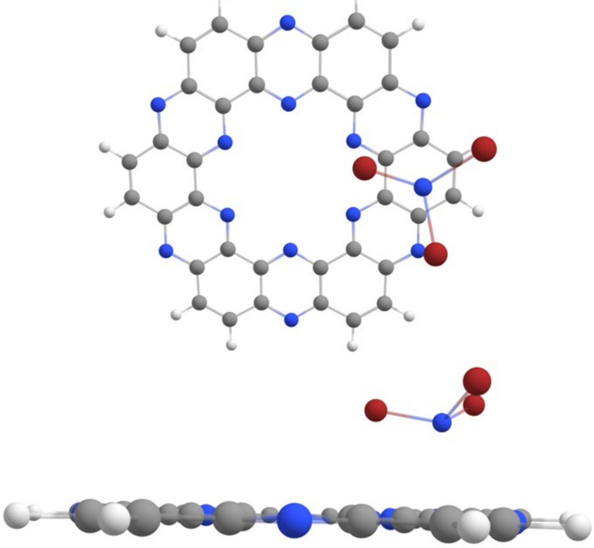

G

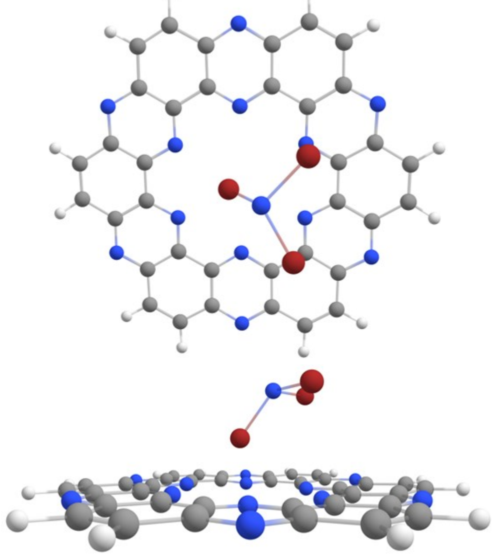

H

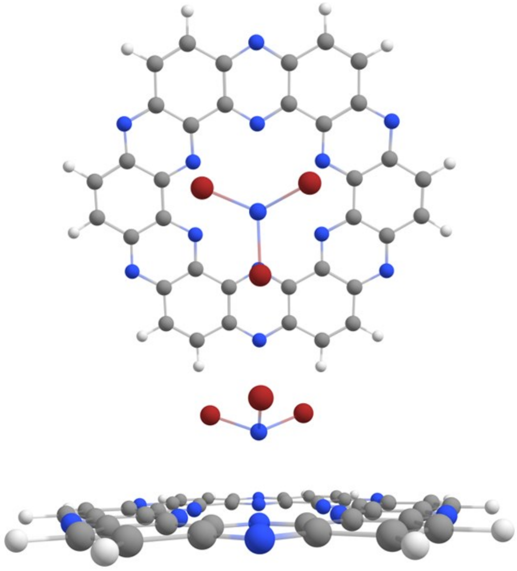

I

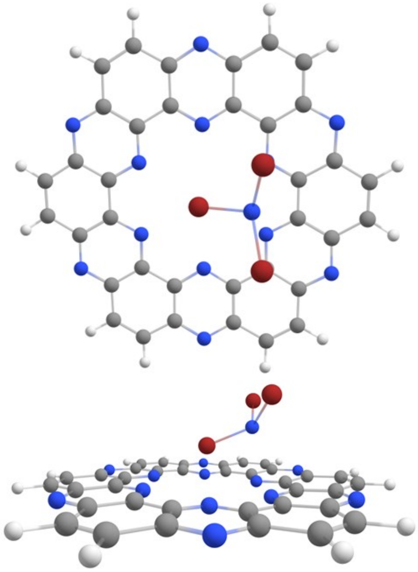

J

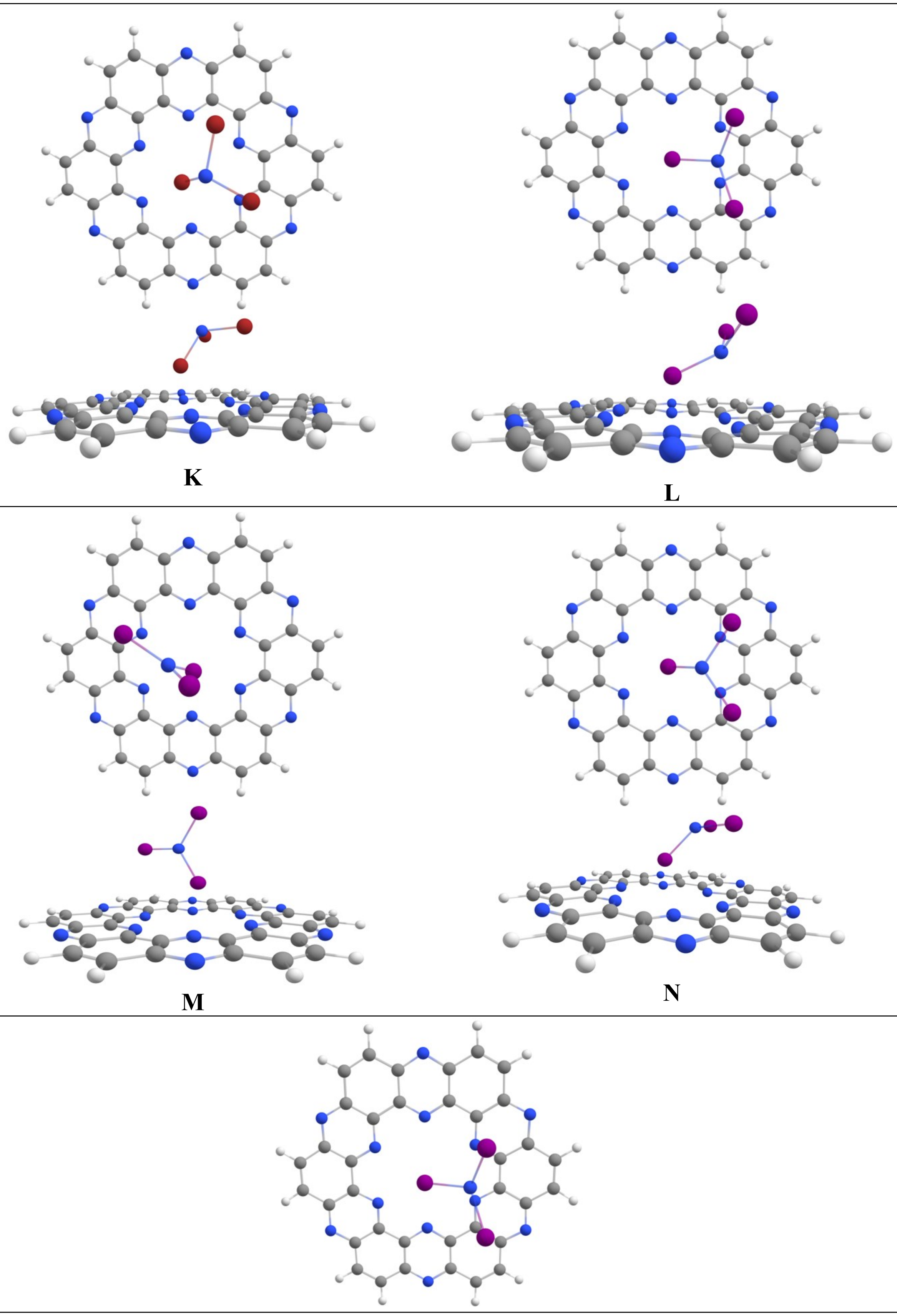
K
L
M
N

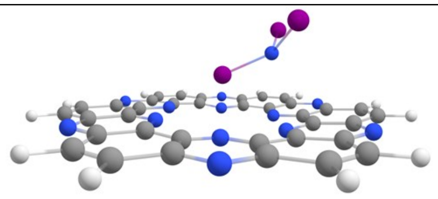

O

Figure S1. Optimized geometries of possible orientation of each analyte over C_2Nsurface at M05-2X (A-E = NCl_3@C_2N, F-J = NBr_3@C_2N and K-O = NI_3@C_2N complexes)

Table S 2. The values of topological parameters of BCPs obtained through QTAIM analysis

**Analytes@C_2N**	**Aanalyte---C_2N**	**ρ**	**$\nabla^2\rho$**	**G (r)**	**V (r)**	**H (r)**	**V(r)/G(r)**	**Eint (kcal/mole)**
NCl_3@C_2N	N1 - Cl9	0.005	0.016	0.0033	-0.0025	0.0007	-0.78	-0.79
	N2 - Cl9	0.006	0.02	0.0041	-0.0032	0.0009	-0.79	-10
	N2 - Cl10	0.006	0.019	0.004	-0.0031	0.0008	-0.79	-0.99
	N3 - Cl9	0.009	0.033	0.0068	-0.0053	0.0015	-0.78	-1.65
	N4 - Cl9	0.011	0.046	0.0091	-0.0067	0.0023	-0.74	-2.11
	N5 - Cl9	0.009	0.033	0.0067	-0.0052	0.0016	-0.77	-1.63
	N6 - Cl9	0.006	0.02	0.0041	-0.0032	0.0009	-0.78	-1.01
	C7 - Cl11	0.005	0.018	0.0037	-0.003	0.0007	-0.8	-0.93
	C8 - Cl11	0.005	0.017	0.0036	-0.0029	0.0007	-0.79	-0.9
NBr_3@C_2N	N1 - Br8	0.006	0.019	0.0037	-0.0028	0.0009	-0.75	-0.87
	C2 - Br9	0.006	0.016	0.0034	-0.0027	0.0007	-0.8	-0.85
	N3 - Br8	0.007	0.024	0.0048	-0.0036	0.0012	-0.75	-1.12
	N4 - Br8	0.006	0.02	0.004	-0.003	0.001	-0.76	-0.95
	N5 - Br8	0.013	0.051	0.0104	-0.0081	0.0023	-0.78	-2.55
	N6 - Br8	0.011	0.039	0.0078	-0.006	0.0018	-0.76	-1.88
	N7 - Br8	0.007	0.023	0.0046	-0.0035	0.0011	-0.75	-1.1
	N7 - Br10	0.006	0.017	0.0035	-0.0027	0.0008	-0.76	-0.84
NI_3@C_2N	N1- I9	0.007	0.02	0.004	-0.003	0.0011	-0.74	-0.93
	N2- I9	0.008	0.026	0.0051	-0.0038	0.0013	-0.75	-1.21
	N2 – I10	0.013	0.043	0.0089	-0.0071	0.0018	-0.8	-2.23
	N3- I9	0.012	0.009	0.0087	-0.0069	0.0018	-0.79	-2.18
	N4 - I9	0.016	0.057	0.0121	-0.0102	0.0019	-0.84	-3.19
	N5- I9	0.008	0.026	0.0052	-0.0039	0.0013	-0.75	-1.23
	C7 – I11	0.005	0.012	0.0024	-0.0018	0.0005	-0.77	-0.57
	C8 – I11	0.005	0.013	0.0025	-0.0019	0.0006	-0.75	-0.59

We used M06-2X and ω-B97XD along with M05-2X to check the trend in interaction energy and electronic properties. The trends of interaction energies and electronic properties predicted by M06-2X/LANL2DZ and ω-B97X/LANL2DZ are similar to those at M05-2X/LANL2DZ for complexes of NX_3@C_2N . Therefore, we feel confident that the results obtained are not dependent on the level of theory.

Interaction energies (kcal/mol) of NX_3@C_2N complexes at different methods using LANL2DZ basis set are:

Bond length (Å)	M05-2X	M06-2X	ω-B97XD
NCl_3@C_2N	-10.85	-11.83	-13.67
NBr_3@C_2N	-13.78	-17.21	-17.04
NI_3@C_2N	-16.33	-20.56	-20.10

The results of energies of HOMO-LUMO gap (E_{H-L} gap) of analytes@C_2N surface at different methods

Methods	M05-2X	M06-2X	ω-B97XD
Analytes	HOMO (eV)	LUMO (eV)	E_{H-L} gap (eV)
C_2N	5.71	5.60	7.05
NCl_3@C_2N	5.58	5.32	6.58
NBr_3@C_2N	5.29	5.04	6.49
NI_3@C_2N	4.15	4.00	5.70
